# Structural equation model-based genome scan for the metabolic syndrome

**DOI:** 10.1186/1471-2156-4-S1-S99

**Published:** 2003-12-31

**Authors:** Catherine M Stein, Yeunjoo Song, Robert C Elston, Gyungah Jun, Hemant K Tiwari, Sudha K Iyengar

**Affiliations:** 1 Department of Epidemiology and Biostatistics, Case Western Reserve University 2500 MetroHealth Dr., R258 Rammelkamp Building, Cleveland, Ohio USA; 2 Department of Biostatistics, 327C Ryals Public Health Building, 1665 University Boulevard, University of Alabama at Birmingham, Birmingham, Alabama USA

## Abstract

**Background:**

The metabolic syndrome is characterized by the clustering of several traits, including obesity, hypertension, decreased levels of HDL cholesterol, and increased levels of glucose and triglycerides. Because these traits cluster, there are likely common genetic factors involved.

**Results:**

We used a multivariate structural equation model (SEM) approach to scan the genome for loci involved in the metabolic syndrome. We found moderate evidence for linkage on chromosomes 2, 3, 11, 13, and 15, and these loci appear to have different relative effects on the component traits of the metabolic syndrome.

**Conclusion:**

Our results suggest that the metabolic syndrome components, diabetes, obesity, and hypertension, are under the pleiotropic control of several loci.

## Background

Metabolic syndrome (MSX) is characterized by the aggregation of several risk factors, including obesity, impaired glucose tolerance, elevated triglyceride levels and blood pressure, and low HDL cholesterol [1]. These factors promote the development of insulin resistance, diabetes, renal insufficiency, and cardiovascular disease. The prevalence of MSX in the United States has been estimated to be 18–24% [2,3]. Studies of the components of MSX demonstrate a substantial contribution of both genetic and environmental factors to disease risk [4].

Methods that account for shared environmental influence on the components of MSX are necessary if the genetic variance is not to be overestimated. Structural equation models (SEM) comprise a valuable method for partitioning the variance into its genetic and shared environmental components. A previous SEM analysis has suggested that the components of MSX are pleiotropically influenced by common genetic factors [5]. Thus, a multivariate analysis of the traits involved may increase power to detect quantitative trait loci (QTL) related to MSX. Although data reductive techniques such as principal component analysis have been performed (e.g. [6,7]), few comprehensive multivariate analyses have been done. In this paper, we analyzed the data from the Framingham offspring study using SEM, which enabled us to model traits related to MSX simultaneously with genetic data and perform a genome scan.

## Methods

### Data

We analyzed data from the offspring cohort of the Framingham Heart Study, provided for Problem 1 of Genetic Analysis Workshop 13. The analysis was performed on all individuals with complete genotypic and phenotypic data for the fifth time point; this sample consisted of 1097 individuals from 381 pedigrees for a total of 1220 sibpairs. The proportion of males and females was roughly equal, and the mean age was 51.33 (± 10.01) years. We modeled MSX using the measurements taken at the fifth time point for systolic blood pressure (SBP), fasting plasma glucose (GLUC), triglycerides (TG), HDL cholesterol (HDL), and body mass index (BMI), which was calculated as weight(kg)/(height(m))^2^. Data from the final time point were used because we hypothesized that the study subjects would best demonstrate any progression to MSX by this time. Both GLUC and TG were log-transformed to reduce skewness. Because the distribution of GLUC was leptokurtotic after log transformation, we further transformed this variable using a generalized modulus power transformation [8]. The shape and scale parameters were estimated using ASSOC [9]. These five phenotypes were covariate-adjusted for age, sex, number of cigarettes smoked per day, number of drinks of alcohol per day, and two-way interaction terms significant at the α = 0.10 level using a stepwise regression model; there were no significant three-way interactions. Standardized residuals were obtained from the regression model and used in the subsequent analyses. Both individual and sibling correlations were estimated using FCOR [9].

### Structural equation model (SEM)

Five adjusted phenotypes were used in multivariate linkage analyses that simultaneously incorporate the phenotypic and genetic marker information into a single SEM, as proposed by Eaves and colleagues [10] and implemented in the Mx package [11]. This approach utilizes the full cross-trait covariance structure between siblings to better separate genetic from within-family environmental effects, which offered several advantages. First, the incorporation of multiple traits in a linkage analysis offers a considerable increase in power, particularly when there is shared environmental variation, even for traits with low heritability [12]. It has been shown that continuous traits have considerably more power for linkage analysis than do binary traits [13]. Finally, this approach is particularly applicable for the measurement of putative "endophenotypes" and is capable of detecting multiple QTL with pleiotropic effects [10].

**Figure 1:**
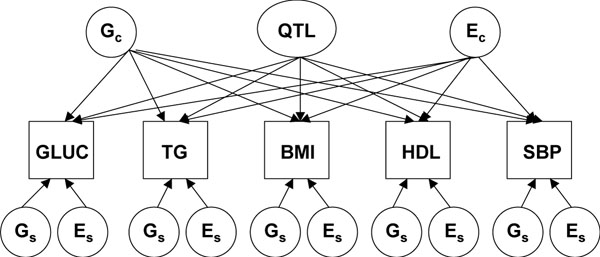
**Path diagram of structural equation model. **G_c _and E_c _are the genetic and environmental influences common to all the components of MSX, respectively, while G_s _and E_s _are the genetic and environmental influences specific to each component.

The methodology is fully described in Eaves et al. [10]. Briefly, using their notation, the sibpair covariance matrix is expected to be



where AA' and EE' are the covariance matrices due to additive genetic and within-family environmental effects, respectively. The information contained about the QTL contained in cross-trait covariances is used to estimate the effects of a putative QTL. The resulting sib-pair covariance matrix is



where k is the number of alleles shared identical by descent (IBD) at the location s, f_k _is the expected proportion of pairs in a random sample sharing k alleles IBD (1/4, 1/2, 1/4), and AA' and EE' are residual additive genetic and environmental effects, after accounting for QQ', the contribution of the QTL effects at the genetic location in question. The SEM including QTL effects is illustrated in Figure 1. The proportion of alleles shared IBD at a genetic locus determines the correlation between the siblings' QTL effects. Thus, two models are evaluated: one with the SEM alone, and another weighted by the IBD probabilities for each sibpair for each genetic locus. Twice the difference between the log likelihood values for these two models gives a likelihood ratio statistic. The contributions of the putative genetic loci to each phenotype are represented as orthogonal factors. If these values (provided in Table 2) are squared, they represent the heritability of the phenotype due to that QTL. We estimated multi-point IBD probabilities using GENIBD [9] at 2cM intervals along the genome, using the parental genotypes from the original Framingham cohort. The linkage analysis was performed using Mx (script available on the Mx home page [14]).

## Results

The individual- and sibling-specific correlations of the adjusted variables are provided in Table 1. This correlation matrix demonstrates that MSX phenotypes cluster within individuals, and that the five phenotypes are correlated between siblings. For each linked region from the SEM-based genome scan, the most significant *p*-values and associated QTL contributions for each phenotype are provided (Table 2). Though no regions attained statistical significance by conventional criteria [15], loci on chromosomes 2, 3, 11, 13, and 15 demonstrated tentative evidence for linkage at markers D2S1353, AFM306yg5, D11S2008, D13S793, D15S165, and D15S642, respectively. Weaker but potential evidence for linkage was seen on chromosomes 4 and 17 at markers D4S1647 and AFM290vc9, respectively. The QTL contributions to each phenotype were estimated. Negative QTL contributions imply that the putative QTL acts to lower the trait value (for example, low HDL increases risk for MSX). For most linked regions, GLUC had the largest contribution, though BMI and SBP were substantially influenced by the QTL as well. Interestingly, TG occasionally had negative QTL contributions and HDL had positive contributions for some loci. Though counterintuitive at first glance, this is most likely an artifact of imprecise estimation. In general, TG and HDL did not yield as strong evidence for linkage as did GLUC, BMI, and SBP.

## Discussion

Previous multivariate analyses of MSX phenotypes have found that insulin and adiposity are highly correlated, and suggested that common genetic factors influence them [5,6]. Utilizing genome-scan data from the Framingham offspring data in conjunction with SEM, we have identified possible loci on several chromosomes that have pleiotropic effects on the component traits of MSX. SEM indicated that the greatest impact of the QTLs was on GLUC, although BMI and SBP were also influenced by these loci, supporting the hypothesis that obesity, glucose intolerance, and hypertension are key factors in MSX [5-7]. Previous factor analyses for MSX are inconsistent with these observations, as glucose and obesity variables frequently loaded together, but there was seldom overlap with blood pressure factors [16].

We observed moderate linkage on chromosomes 2, 3, 4, 11, 13, and 15, although the relative effect of these putative QTL on each MSX phenotype appears to differ. Because the literature related to linkage signals for MSX components is vast, we have summarized reports within a 30cM window of our linkage signals [17]. Though we conducted a truly multivariate analysis of MSX, our results can be considered confirmations of these previous reports. Body size [7], diabetes mellitus [18], and insulin resistance [19] have been linked to chromosome 11; this region also contains several candidate genes, including insulin (OMIM 17673), *SHIP2 *(OMIM 600829), and the uncoupling protein 2 (OMIM 601693) [20]. An adiposity-insulin factor attained suggestive linkage to chromosome 2 [6], and a separate study found linkage to BMI 20 cM away [21]. Insulin resistance [19], BMI [19], and high blood pressure [22] have been linked to chromosome 3. Leptin levels have been linked to the 20-cM location on chromosome 15 [21]. Finally, *IRS-2*, a diabetes candidate gene, has been mapped to chromosome 13 (102 cM) [23].

Given the multifactorial nature of MSX, the method of linkage analysis most suitable is one that incorporates all available trait information. Here we use SEM with multivariate data to increase power, account for shared sibling environment within a sib-pair linkage analysis, and depict the combined effects of the components of MSX simultaneously. This approach is uniquely capable of estimating the contribution of each phenotype to the QTL, which can illustrate facets of MSX biology. Another advantage of our approach is the direct use of quantitative traits. Currently, there are two different clinical definitions of MSX [2], which require the presence of at least two (or three) of five characteristics. By analyzing the full quantitative scale for each MSX component, not only do we gain power statistically, but we also avoid classifying individuals together who have moderate to extreme trait values. As observed in Table 1, these traits are not highly correlated with each other, but our genome scan results suggest that they may be influenced by common genetic factors.

There are, however, limitations to these analyses. Although there were data available on blood pressure medication, there were no data on diabetes medication. Since both were not available, we did not adjust our quantitative variables for these covariates. Because of missing genotype and/or phenotype data, approximately 700 sibpairs were excluded from this analysis. Though our sample size was still substantial, it is unknown how these sibpairs might have increased power. There is an upward bias in the estimation of the QTL contributions. The sum of the squared QTL contributions to GLUC for all distinct linked regions is greater than 1, which is not possible if these values are interpreted to be QTL-specific heritabilities. Also, when partitioning genetic and shared-environmental components of variance, gene×environment interaction was unaccounted for, and allowing for such interaction would only exacerbate the discrepancy. Finally, in the SEM, all sibpairs were treated as though they were independent. It is unclear how to handle larger sibships within the SEM; however, the average sibship size is less than three, so this should not have a great impact on the analysis.

Another point worth revisiting is our approach to non-normal data. The SEM assumes multivariate normality and, with increasing sample size, departure from this assumption will affect the likelihood ratio test adversely. In our analysis, the GLUC variable was transformed using a generalized modulus power transformation [8] to remove skewness and kurtosis. Though the departure of this variable from normality may be regarded as biologically based [24], a leptokurtotic distribution leads to an inflated type I error rate [24], and the scale of measurement may drastically affect results of linkage analysis [25]. In fact, when this analysis was performed without transformation, most of the linkage results were more significant, and the QTL contributions were incorrectly inflated (data not shown). After transformation, the relative effects on the other MSX traits increased, indicating that the variance was partitioned more evenly when univariate normality of the five traits was attained.

## Conclusions

A number of loci appear to be linked to MSX, most notably regions on chromosomes 2, 3, 11, 13, and 15. Diabetes, obesity, and hypertension were most influenced by these loci. These regions appear to influence the components of MSX in different ways, and they warrant further analysis, both in reference to the metabolic syndrome itself and to other disorders associated with insulin-resistance.

## Figures and Tables

**Table 1:** Individual- and sibling-specific correlations.

	GLUC	TG	BMI	HDL	SBP
GLUC	**0.2306**	0.2118	0.3187	-0.1784	0.2491
TG	**0.0670**	**0.2247**	0.2917	-0.5326	0.3316
BMI	**0.1130**	**0.0973**	**0.2921**	-0.2883	0.2206
HDL	**-0.0276**	**-0.1911**	**-0.0624**	**0.2622**	-0.1024
SBP	**0.1143**	**0.0786**	**0.1027**	**-0.0620**	**0.1825**

Unbolded values are individual-specific correlations; bolded values are sibling-specific correlations.

**Table 2:** Linked regions for MS identified using SEM.^A^

			QTL contribution				
			
Region	Location (cM)	*p*-Value	GLUC	TG	BMI	HDL	SBP
2q24.2	165	0.003	0.4633	0.1344	0.3305	-0.1086	0.3044
3p21.31-3p13	71–112	0.003	0.4537	0.2567	0.4101	-0.0987	0.2894
4q24.2	88	0.024	0.2258	-0.0360	0.3348	-0.0823	0.4879
11p15.5-11q23.3	9–113	0.003	0.3352	0.2877	0.3639	-0.0588	0.3417
12q14.3-12q15	78	0.029	0.2242	0.0290	0.3839	0.0366	0.4476
13q32	76	0.009	0.3393	0.1286	0.2361	0.0127	0.3627
15q11.2-15q31.1	5–20	0.005	0.3221	-0.0547	0.0433	-0.2067	0.2927
15q26.3	122	0.011	0.4494	0.1068	0.2088	-0.0454	0.3349
17p12	31–32	0.030	0.2102	0.1363	0.2268	-0.0275	0.4786

^A^Marker names are provided within the text.
